# Does Resin Cement Type and Cement Preheating Influence the Marginal and Internal Fit of Lithium Disilicate Single Crowns?

**DOI:** 10.3390/ma15020424

**Published:** 2022-01-06

**Authors:** Nourhan Samy, Walid Al-Zordk, Ahmed Elsherbini, Mutlu Özcan, Amal Abdelsamad Sakrana

**Affiliations:** 1 Fixed Prosthodontics Department, Faculty of Dentistry, Mansoura University, Mansoura 35516, Egypt; nourhan.samy2010@gmail.com (N.S.); walidwa@gmail.com (W.A.-Z.); 2 Fixed Prosthodontics Department, Faculty of Dentistry, Horus University, Damietta 34511, Egypt; 3 Mansoura General Hospital, Mansoura 35511, Egypt; dr.ahmedelsherbini6@gmail.com; 4 Center for Dental and Oral Medicine, Division of Dental Biomaterials, Clinic for Reconstructive Dentistry, University of Zürich, 8032 Zürich, Switzerland; mutluozcan@hotmail.com

**Keywords:** resin cement, preheating, marginal and internal adaptation, IPS e.max Press

## Abstract

This paper assesses the effect of cement type and cement preheating on the marginal and internal fit of lithium disilicate single crown. Methods: 40 maxillary premolars were selected, restored with lithium disilicate single crowns. Teeth were randomly assigned into four groups (n = 10) based on cement type (Panavia SA or LinkForce) and preheating temperature (25 °C or 54 °C). After fabrication of the restoration, cements were incubated at 25 °C or 54 °C for 24 h, and each crown was cemented to its corresponding tooth. After 24 h, all specimens were thermally aged to (10,000 thermal cycles between 5 °C and 55 °C), then load cycled for 240,000 cycles. Each specimen was then sectioned in bucco-palatal direction and inspected under a stereomicroscope at x45 magnification for marginal and internal fit evaluation. The data were statistically analyzed (significance at *p* ≤ 0.05 level). **Results:** At the mid-buccal finish line, mid-buccal wall, palatal cusp, mid-palatal wall, mid-palatal finish line, and palatal margin measuring points, there was a significant difference (*p* ≤ 0.05) between the lithium disilicate group cemented with Panavia SA at 25 °C and the group cemented with LinkForce at 25 °C, while there was no significant difference (*p* > 0.05) at the other points. At all measuring points, except at the palatal cusp tip (*p* = 0.948) and palatal margin (*p* = 0.103), there was a statistically significant difference (*p* ≤ 0.05) between the lithium disilicate group cemented with Panavia SA at 54 °C and the group cemented with LinkForce at 54 °C. Regardless of cement preheating, statistically significant differences were found in the buccal cusp tip, central groove, palatal cusp tip, and mid-palatal wall (*p* ≤ 0.05) in the lithium disilicate group cemented with Panavia SA at 25 °C and 54 °C, as well as the mid-palatal chamfer finish line and palatal margin in the LinkForce group cemented with Panavia SA at 25 °C and 54 °C. At the other measurement points, however, there was no significant difference (*p* > 0.05). **Conclusions:** The type of resin cement affects the internal and marginal fit of lithium disilicate crowns. At most measuring points, the cement preheating does not improve the internal and marginal fit of all lithium disilicate crowns.

## 1. Introduction

Ceramic restorations are frequently used in daily dental practice because of their superior esthetic characteristics, good mechanical properties, biocompatibility, and long-term stability [1]. For proper selection of ceramic restoration, the choice has been increasingly oriented toward lithium disilicate [2]. Lithium disilicate is classified as a glass-ceramic, in the class of particle-filled glass materials [3]. Heat-pressing or CAD/CAM processing methods are used to make lithium disilicate restorations. The mechanical characteristics of these restorations are influenced by the block composition and the production procedure [4,5]. Because of its superior esthetics, adhesive characteristics, ability to preserve tooth structure, and good fracture resistance, lithium disilicate materials have been widely promoted [6,7,8].

The major role of the cement is to seal the area between the restoration and prepared tooth, reduce the micro-leakage, preventing it from being loosened and dislodged [9]. To establish retention of a restoration to tooth preparation or implant abutment and maintain its integrity, dental cement should ideally meet particular biological, physical, mechanical, and handling features [10,11,12]. The dual-cure resin cement appears to have improved bond strength and hardness when compared to self-cure resin cement [13]. The self-adhesive resin cements were created to make the cementation step easier since these cements do not require the tooth to be conditioned or the restoration to be pre-treated [14]. Self-adhesive resin cements have a high viscosity, which can rapidly increase after an acid–base reaction [15].

Residual monomers and unreacted photo-initiators from incomplete monomer conversions can leak into the oral cavity, resulting in allergic responses and bacterial build-up around the restorations [16]. Because the mechanical properties and polymerization kinetics of polymers are altered by the heat degree, it was advised to warm-up refrigerator-stored resin cements to at least room temperature before clinical use and pre-heating to 60 °C to reinforce the bonding potential of the tested resin cement [17,18]. Increased temperature during polymerization of dual-cure resin cements raised the degree of conversion, according to a study that investigated the influence of temperature on the degree of conversion and working duration of dual-cured resin cements exposed to varied curing conditions [19].

An essential measurement for evaluating restorative fit is the fit discrepancy between the interior surface of the restoration and the prepared tooth [20,21,22,23]. Gingival health is ensured by the good marginal fit of the restoration, which results in less cement dissolution, decreases the possibility of discoloration, and reduced the potential of recurrent caries [24]. Internal fit enhances mechanical qualities such as retention and fracture resistance, whereas poor internal fit can result in compromised retention and weakening of the restored tooth [25,26,27]. Many factors influence the marginal and internal fit, including the ceramic type, cement type, geometry of tooth preparation, material handling, curing method, and the procedure employed to remove excess cement [28]. The fit of a restoration could be verified and evaluated by many methods such as direct viewing of the margin, cross-sectioning, and replica technique [29,30]. Therefore, the purpose of this investigation was to assess the influence of adhesive cement type and cement preheating on the fit of lithium disilicate single crowns. The first null hypothesis was that the type of resin cement will not affect the fit of lithium disilicate crowns. The second null hypothesis was that the pre-heating of adhesive resin cement will not affect the fit of lithium disilicate crowns.

## 2. Materials and Methods

Forty sound maxillary premolars teeth of comparable dimensions, extracted for orthodontic purposes, were collected for this in vitro study. These teeth were examined under proper lightening to ensure there freed from caries, cracks and, fractures. Teeth were disinfected in sodium hypochlorite solution bleach (Clorox Bleach, Clorox Egypt CO., Cairo, Egypt) for one week [30]. To avoid dehydration, teeth were kept in standardized saline solution (Sodium Chloride BP 0.9 percent, Fibco, Alexandria, Egypt) at room temperature until use. The included teeth dimensions, as measured using a digital caliper (150 mm/6 in, American spares, Colorado Springs, CO, USA), were 5.5 ± 0.5 mm occluso-cervical, 7 ± 0.5 mm mesio-distal, and 9 ± 0.5 mm bucco-palatal. Table 1 shows how the teeth were classified into four groups based on the type of resin cement used (either Panavia SA cement plus or G-Cem LinkForce cement) and cement preheating (either at 54 °C or 25 °C).

**Figure 1:**
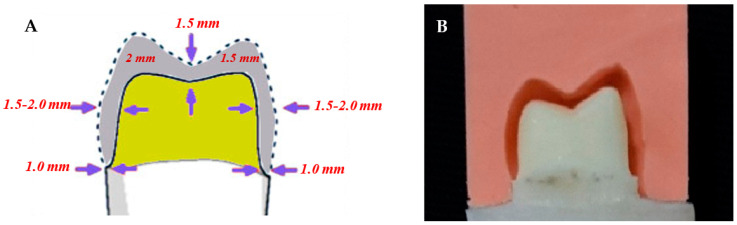
(**A**) Illustration of tooth preparation for lithium disilicate crown; (**B**) Proximal view for prepared tooth inside the putty index.

Prior to tooth preparation, a putty index was made for each tooth using a vinyl polysiloxane impression material (Imflex Putty, Meta Biomed Co., Cheongju-si, Korea). CAD-CAM technology was used for teeth preparation to control tooth reduction [31]. Each tooth was prepared using a high-speed handpiece and a dental surveyor (Dentalfarm A3006 B manual surveyor, Turin, Italy) with preparation dimensions corresponding to the lithium disilicate restorative material (1 mm chamfer margin, 2 mm palatal cup reduction, 1.5 mm buccal cusp reduction and 6-degree axial wall taper). The amount of tooth reduction was evaluated using the pre-preparation putty index (Figure 1).

Each tooth was scanned using the CAD-CAM scanner (ceramill Map 400 optical scanner, Amann Girrbach GmbH, Koblach, Austria) after being coated with anti-reflection scan powder (Telescan white, DFS Diamon GmbH, Riedenburg, The Netherlands). The crown design was created with CAD-CAM software (Ceramill Mind, Amann Girrbach GmbH, Koblach, Austria) and an 80-μm cement space was chosen, after which a pattern was 3D printed (Phrozen shuffle, phrozen Technology, Hsinchu, Taiwan) using a printer resin (FTD Dentifix-3D LR, 3D printing resin, Lumi industries, Montebelluna, Italy). The resin patterns were sprued and invested, then the pressing procedures were performed following the instructions of the manufacture using a lithium disilicate ingots (IPS e.max Press, Ivoclar Vivadent, Schaan, Liechtenstein). Then, each crown was finished and cleaned before being subjected to crystallization and glaze firing following the instructions of the manufacture.

For bonding, the intaglio surfaces of the crowns were etched (9% Porcelain etch, Ultradent, South Jordan, UT, USA) for 20 s. The resin cements were preheated (25 °C, 54 °C) using an incubator (Series BD model 56, Standard Incubators, BINDER GmbH, Tuttlingen, Germany). The abutments in the LR and LH groups were pre-treated with the bonding agent (G-Premio BOND, GC Corp., Tokyo, Japan) for 15 s before being light-cured (LED curing light, Guilin Woodpecker Medical Instrument Co., Guilin, China) for 20 s. The corresponding resin cement was dispensed in the intaglio surface of each crown for all specimens. The crown was then mounted on its corresponding tooth and held under a load of 10 N throughout polymerization (Instron Universal testing machine, 3345, Norwood, MA, USA) [32]. To enable the removal of excess cement, an initial light curing for 3 s was conducted, then the final curing was done for 20 s on each side, according to the manufacturer’s recommendations.

Thermal aging (Thermo-cycler SD Mechatronic GmbH, Munich, Germany) was performed on all specimens for 10,000 cycles (5 °C/55 °C). Then, all specimens were subjected to 240,000 load cycles (chewing simulator CS4.4, SD Mechatronic GmbH, Munich, Germany) with 50-N load at 60 mm/s [33].

All the specimens were sectioned centrally from the buccal to palatal direction into two sections. Linear precision cutting micro-saw (Isomet 4000 linear cutting microsaw, Buehler, Germany) was used.

**Figure 2:**
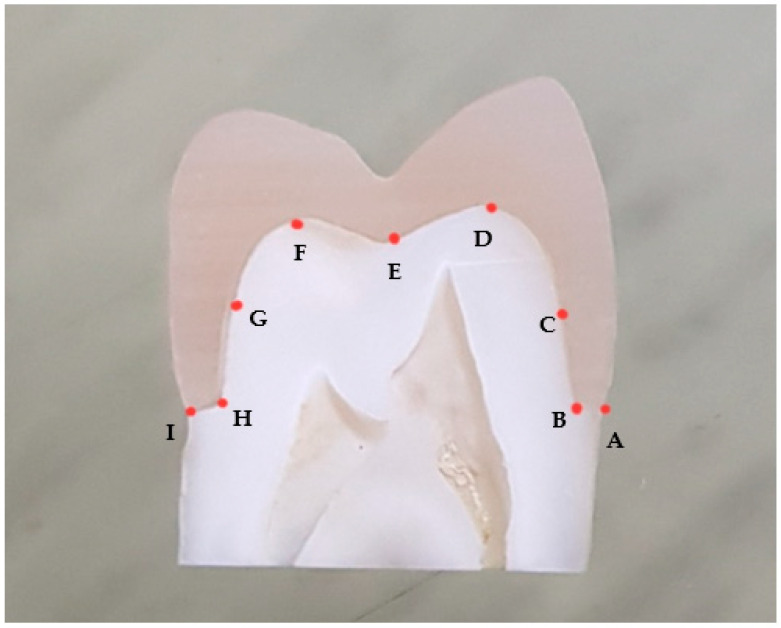
Points of measurement of marginal and internal fit; (**A**) buccal margin, (**B**) buccal chamber finish line, (**C**) mid-buccal axial wall, (**D**) Buccal cusp tip, (**E**) central groove, (**F**) palatal cusp tip, (**G**) mid-palatal axial wall, (**H**) palatal chamfer finish line, (**I**) palatal margin.

Measurement of marginal and internal fit: The cut sections were examined under a stereomicroscope (Olympus SZ 61, Tokyo, Japan) at 45 magnification to measure the marginal and internal fit at nine locations (buccal margin, mid-buccal finish line, mid-buccal axial wall, buccal cusp tip, central groove, palatal cusp tip, mid-palatal axial wall, mid-palatal finish line, palatal margin) as shown in Figure 2. Then the measurements data were tabulated and statistically analyzed.

Data were analyzed a software (SPSS V 22.0, IBM SPSS corp., Armonk, NY, USA). The normality of data was checked using the Shapiro–Wilk test. A two-way ANOVA test was performed to detect the effect of the two independent variables and Student’s *t*-test was used to compare two independent groups.

## 3. Results

There was a statistically significant difference between lithium disilicate crowns cemented with Panavia SA cement at 25 °C and that preheated at 54 °C at the buccal margin (t = 2.69, *p* = 0.02), buccal cusp tip (t = 2.77, *p* = 0.013), central groove (t = 3.45, *p* = 0.003), palatal cusp tip (t = 4.91, *p* < 0.001), and mid-palatal axial wall (t = 6.38, *p* < 0.001); however, no significant differences at other points of measurement. Additionally, there was a significant difference between lithium disilicate groups cemented with LinkForce cement at 25 °C and that cemented with LinkForce cement preheated at 54 °C at all points of measurement except at the buccal margin (t = 1.69, *p* = 0.107), buccal finish line (t = 0.958, *p* = 0.351), and mid-buccal axial wall (t = 0.253, *p* = 0.803).

There was a statistically significant difference between lithium disilicate crowns cemented with Panavia SA cement at 25 °C and LinkForce cement at 25 °C at all measurement points except at the buccal margin (t = 1.43, *p* = 0.171) and central groove (t = 1.55, *p* = 0.138). In addition, there was a significant difference between lithium disilicate crowns cemented with Panavia SA cement preheated at 54 °C and LinkForce cement preheated at 54 °C except at the palatal margin (t = 1.72, *p* = 0.103) and palatal cusp tip (t = 0.07, *p* = 0.948). Table 2.

**Figure 3:**
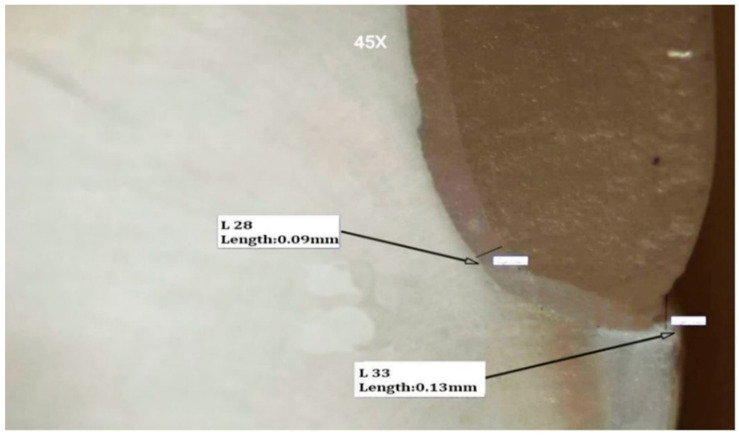
Stereomicroscopic view (45X) represents marginal and internal fit of a sectioned specimen at buccal margin and buccal finish line.

**Figure 4:**
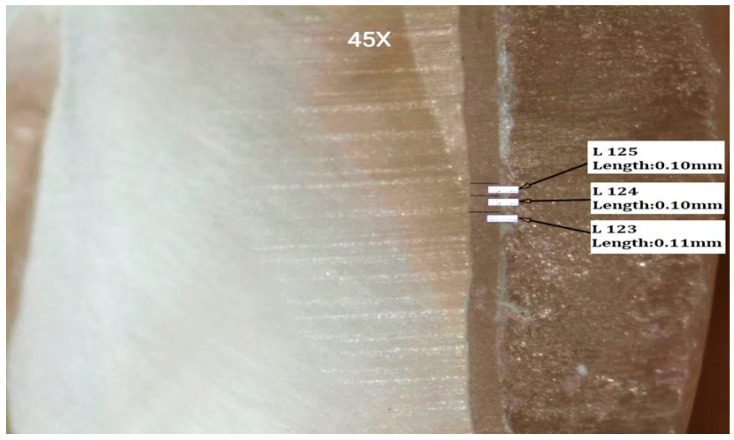
Stereomicroscopic view (45X) represents marginal and internal fit of a sectioned specimen at mid buccal axial wall.

**Figure 5:**
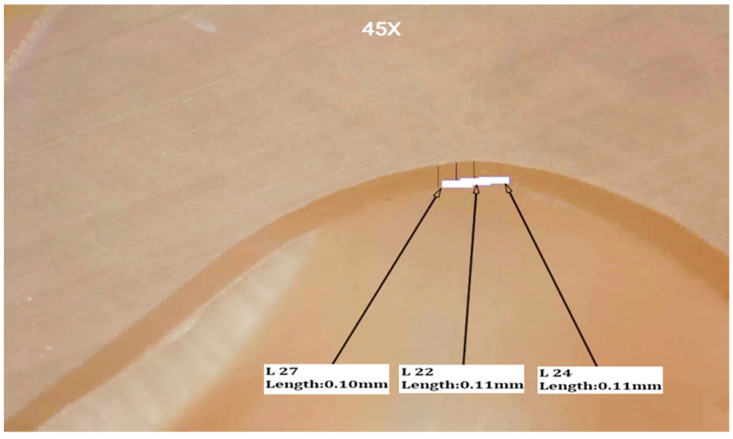
Stereomicroscopic view (45X) represents marginal and internal fit of a sectioned specimen at buccal cusp tip.

**Figure 6:**
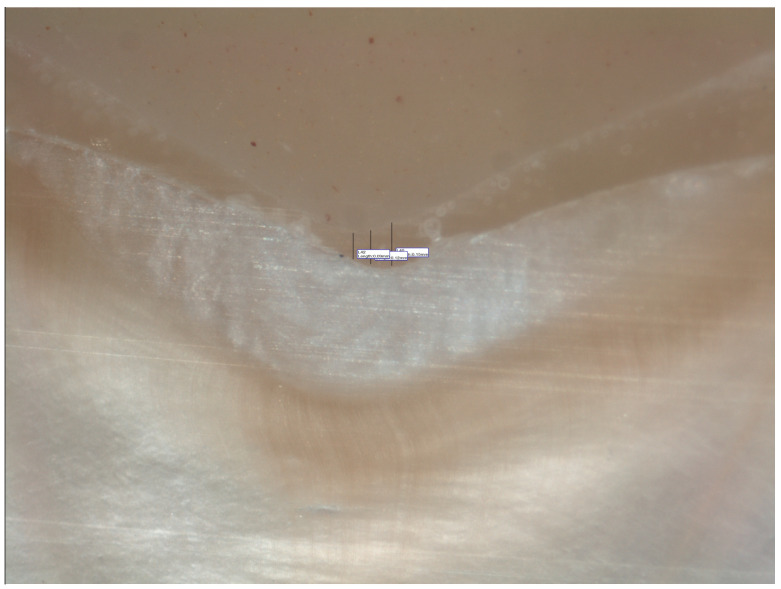
Stereomicroscopic view (45X) represents marginal and internal fit of a sectioned specimen at central groove.

The two-way ANOVA test revealed that there was a statistically significant combined effect of changing cement type and cement preheating on buccal margin (with 40.4%), buccal cusp tip (with 92.1%), central groove (with 68.7%), mid palatal axial wall (with 64.3%), mid palatal finish line (with 78.2%), palatal margin (with 27.8%) of each point is affected by these factors. A representative stereomicroscopic images of marginal and internal fit of specimens are shown in Figure 3, Figure 4, Figure 5 and Figure 6.

## 4. Discussion

The aim of this study was to evaluate the influence of resin cement type and preheating on the marginal and internal fit of lithium disilicate restoration. Both tested hypotheses were rejected as the results revealed that the resin cement type and cement preheating significantly affects the marginal and internal fit of lithium disilicate single crowns.

The maxillary first premolars were chosen for the present study, with special attention made to the selection of teeth with comparable sizes [34]. Dual-cured resin cements are suitable for the cementation of ceramic restorations because of the combined benefits of both light and chemical cure mechanisms to ensure adequate and complete polymerization of the material even when light activation is insufficient [35]. An effective polymerization process can achieve a higher degree of monomer conversion in resin materials [36]. However, polymerization stress represents a concern with the use of dual-cured resin cements. Delaying the start of light curing (the time from the start of cement mixing and seating of the restoration) could lead to a reduction in these polymerization stresses [37]. In the current study, a short initial light cure was performed to facilitate removal of excess cement, then the final curing was performed. However, the tack-curing may interfere with the polymerization. Other studies found that the initial light curing had no influence on the mechanical properties of the cement [38,39]. Furthermore, in our investigation, preheating resin cement to 54 °C was chosen because previous studies suggested that preheating to 55–60 °C could induce pulp damage and breakdown in some types of resin cement [17,18,19].

Because it has a better vertical marginal gap than its comparable CAD-CAM materials, pressable lithium disilicate was chosen [40]. A study compared the marginal fit of lithium disilicate crowns generated using the hot-press technique to those fabricated by the CAD-CAM approach and concluded that the pressed restorations produce improved marginal fit compared to the CAD/CAM restorations [2].

Standardized tooth preparation was done utilizing CAD-CAM technology for increased control over tooth preparation, which offers numerous advantages over hand fabricated models [41]. Clinical variables influencing the marginal fit of ceramic restorations include tooth preparation geometry, type of finish line, cement type, and ceramic manufacturing technique and material [42].

Artificial ageing is an experimental strategy for determining the clinical behavior of a restorative material by simulating oral environmental circumstances extra-orally [43]. With thermal aging, repeated stresses induce of contraction and expansion, resulting in the propagation of cracks and fractures, loss of retention, the creation of cracks at interfaces, and degradation [44]. In addition, the applied aging may alter the marginal and internal fit, as reported by Dessouky et al. [45], who found that artificial aging increases the vertical marginal discrepancy by thermo-mechanical stress.

The cross-sectioning technique was adopted in this work to allow direct measurement of the internal and marginal adaption in both vertical and horizontal planes under the microscope, reducing the possibility of repositioning errors. Several studies employed the cross-sectioning methodology to assess fit precision and found it to be more reliable than other methods [46]. However, some data may be lost during sectioning procedures as a result of this technique [30]. Additionally, micro-computed tomography provides excellent non-destructive tool for analysis of the internal and marginal adaption [47].

The results of the current study revealed a statistically significant effect of preheated cements on the marginal and internal fit of lithium disilicate crowns. This is in line with a prior work by Elsayed [48], which discovered that preheated resin composites increase adaptability and reduce interfacial gaps. Aygun et al. [49] also found that preheating the self-adhesive G-Cem cement to 37 °C improved marginal adaption compared to the application at 25 °C and 54 °C. Restorations cemented using etch-rinse cement (Variolink N high viscosity cement) mixed at 25 °C, 54 °C, and 37 °C performed better clinically than those cemented with self-adhesive G-Cem at the same temperatures.

Two types of resin cements were employed in the current study: Panavia SA Cement Plus self-adhesive resin cement and G-CEM LinkForce adhesive resin cement. On the marginal and internal fit of lithium disilicate crowns, there was a considerable difference between the utilized cements. This finding is partially in line with Raafat [50], who looked into the influence of CAD/CAM restorations, heat-press technique, and resin cement type on the marginal and internal adaptation of all ceramic IPS e.max crowns. The solubility of the cement used plays an important role in providing better restoration fit [51]. Because the insoluble resin cement absorbs water, it may aid in the relaxation of internal tensions induced by polymerization shrinkage, lowering the risk of inter-facial failure during thermocycling [52].

The current study found that cement type and cement preheating had a substantial effect on specific points of measurement among the analyzed groups, but not on other points of measurement. This could be due to what is known as the overshoot phenomenon. Overshoot phenomena, which replicate virtual peaks on the borders, might cause internal gaps to widen at particular spots [53].

As a limitation of this study, only one type of ceramic materials was used with only two types of resin cements. The differences in the selected natural teeth, since it was difficult to select a perfect match for all selected teeth. This study was performed to simulate limited period in the oral environment. Cross-sectioning technique was used in a destructive method. Thus, other technique as micro-computed tomography which provides a non-destructive tool for analysis of the adaption is required. Clinical studies are needed to test the effectiveness of preheating on the clinical performance of full-coverage restorations.

## 5. Conclusions

The type of resin cement affects the internal and marginal fit of lithium disilicate crowns. At most measuring points, the cement preheating does not improve the internal and marginal fit of all lithium disilicate crowns.

## Figures and Tables

**Table 1:** The study groups.

Group	Code
Restoration cemented using Panavia SA at 25 °C	PR
Restoration cemented using Panavia SA at 54 °C	PH
Restoration cemented using LinkForce at 25 °C	LR
Restoration cemented using LinkForce at 54 °C	LH

**Table 2:** Means and standard deviations of the marginal and internal fit (μm) of the studied groups.

Points of Measurements	Panavia SA Cement		LinkForce Cement	
	25 °C	54 °C	25 °C	54 °C
Buccal margin	107.10 ± 26.15 ^A^	79.9 ± 18.38 ^B^	122.90 ± 23.35 ^B,C^	149.30 ± 43.26 ^A,C^
Mid-buccal chamfer	106.5 ± 16.34 ^A^	104.70 ± 19.71	129 ± 22.69 ^B^	141 ± 32.47 ^A,B^
Mid-buccal axial	109.20 ± 13.07 ^A,B^	119.20 ± 20.69 ^A,C^	152.40 ± 28.65 ^C,D^	149.20 ± 27.96 ^B,D^
Buccal cusp tip	126.50 ± 24.50 ^A,B^	151.50 ± 14.73 ^C^	131.40 ± 15.32 ^A,D^	273.10 ± 14.20 ^B,C,D^
Central groove	227.0 ± 40.84 ^A,B^	177 ± 20.72 ^C^	204.40 ± 21.17 ^A,D^	286 ± 18.98 ^B,C,D^
Palatal cusp tip	147.90 ± 26.48 ^A,B^	210.30 ± 30.23 ^C,D^	108.90 ± 10.21 ^A,C^	208.60 ± 76.02 ^B,D^
Mid-palatal axial	195.90 ± 43.7 ^A,B^	103.50 ± 13.55 ^A,C^	112.10 ± 20.29 ^B,D^	197.10 ± 42.45 ^C,D^
Mid-palatal chamfer	109.5 ± 8.96 ^A,B^	121.50 ± 19.01 ^A,C^	140 ± 25.49 ^D^	202 ± 18.14 ^B,C,D^
Palatal margin	157.70 ± 51.05 ^A^	120.90 ± 28.99 ^A,B^	92.90 ± 21.98	148.80 ± 42.43 ^B^

Note: Means with similar superscripted letters in the same row denote significant difference between groups by post hoc Tukey test.

## Data Availability

The data presented in this study are available on request from the corresponding author A.A.S.
